# A Retrospective Study on Biliary Cooling During Thermal Ablation of Central Liver Tumors in Taiwan

**DOI:** 10.3390/cancers17111859

**Published:** 2025-05-31

**Authors:** Yi-Chun Chou, Chih-Wei Tseng, Ping-Hung Ko, Tsung-Hsing Hung, Hsing-Feng Li, Kuo-Chih Tseng, Ching-Sheng Hsu, Chih-Ying Wang

**Affiliations:** 1 Division of Gastroenterology, Department of Internal Medicine, Dalin Tzu Chi Hospital, Buddhist Tzu Chi Medical Foundation, Chia-Yi 62247, Taiwan; swankyblade@yahoo.com.tw (Y.-C.C.); dm650504@tzuchi.com.tw (C.-W.T.); dm166239@tzuchi.com.tw (P.-H.K.); dm512373@tzuchi.com.tw (T.-H.H.); dm752049@tzuchi.com.tw (H.-F.L.); s121387@tzuchi.com.tw (K.-C.T.); 2 School of Medicine, Tzu Chi University, Hualien 97004, Taiwan; 3 Center for Digestive Medicine, Taichung Tzu Chi Hospital, Buddhist Tzu Chi Medical Foundation, Taichung 42743, Taiwan; 4 Department of Medical Research, Taichung Tzu Chi Hospital, Buddhist Tzu Chi Medical Foundation, Taichung 42743, Taiwan; 5 School of Post-Baccalaureate Chinese Medicine, Tzu Chi University, Hualien 97004, Taiwan; 6 Department of Medical Research, Dalin Tzu Chi Hospital, Buddhist Tzu Chi Medical Foundation, Chia-Yi 62247, Taiwan; dl34170@tzuchi.com.tw

**Keywords:** biliary cooling, cool saline solution, thermal ablation, central liver tumor, nasobiliary drainage

## Abstract

**Background:** Thermal ablation of centrally located liver tumors carries an increased risk of bile duct injury due to their proximity to the biliary tree. We aim to evaluate whether biliary cooling using a nasobiliary tube can effectively mitigate bile duct injury during the ablation process. **Methods:** We retrospectively analyzed the data of 322 patients who underwent thermal ablation at Dalin Tzu Chi Hospital from July 2020 to June 2023 and identified those who received prophylactic biliary cooling during thermal ablation for central liver tumors. Data including demographics, tumor characteristics, procedural details, and clinical outcomes were analyzed. **Results:** Among the 322 patients who underwent thermal ablation, 9 with central liver tumors received prophylactic biliary cooling. The median distance between the tumor and the central bile duct was 1 mm (range: 0–4 mm), the temperature of the cold normal saline was 4 °C, and the mean volume of normal saline infused was 150 mL (range: 100–200 mL). Complete ablation was achieved in all patients in a single session without any biliary injury. One patient developed acute cholangitis after ENBD placement, which resolved with antibiotic therapy. **Conclusions:** Biliary cooling with 4 °C cold saline through a nasobiliary tube can improve the safety and effectiveness of thermal ablation for central liver tumors.

## Simple Summary

This retrospective study evaluates the clinical outcomes of endoscopic nasobiliary drainage (ENBD)-assisted biliary cooling during thermal ablation of central liver tumors. The findings indicate that cold saline perfusion via ENBD can effectively prevent biliary thermal injury during both radiofrequency ablation (RFA) and microwave ablation (MWA), enabling complete tumor eradication with minimal complications. This study also outlines practical endoscopic considerations and safety strategies to facilitate the integration of biliary cooling into interventional oncology protocols.

## 1. Introduction

Thermal ablation techniques, including radiofrequency ablation (RFA) and microwave ablation (MWA), have emerged as minimally invasive treatment modalities for both primary and metastatic liver tumors [1]. These methods are endorsed by international guidelines as curative options, particularly for hepatocellular carcinoma (HCC) in selected patients [2,3]. They offer a minimally invasive alternative to surgical resection, especially in patients with compromised liver function or multiple comorbidities [1,2,3]. However, thermal damage to adjacent structures, particularly the biliary system, remains a major concern. Heat-induced injury to the central bile ducts can result in complications such as biliary strictures, cholangitis, biloma formation, and bile duct necrosis, which may adversely affect patient outcomes [4,5,6].

To mitigate these risks, biliary cooling techniques have been introduced. This involves the perfusion of chilled saline, or 5% dextrose in water, into the bile ducts via nasobiliary tubes, transcystic catheters, or direct choledochal cannulation during thermal ablation [7]. The cooling mechanism prevents excessive temperature elevation in the bile ducts, thereby reducing the risk of thermal injury while maintaining the effectiveness of tumor ablation [6,8,9,10,11,12,13,14]. Notably, a retrospective study demonstrated a biliary injury rate of 46% among patients with tumors adjacent to the central bile duct when biliary cooling was not applied, compared to a significantly reduced rate of 2.5% with intraductal chilled saline perfusion [8]. Additionally, a systematic review and pooled analysis suggested that the overall rate of biliary complications could be as low as 2% with biliary cooling during RFA of liver lesions close to central bile ducts [7].

Despite promising results, further investigation into biliary cooling techniques is essential due to several critical gaps in the existing literature. The majority of existing studies are small-scale, retrospective, and single-center, limiting the applicability of their findings [1,7,15]. Concerns persist that excessive cooling may compromise the efficacy of thermal ablation, potentially increasing local tumor recurrence rates [9]. The tumor recurrence rate at the RFA target site during follow-up may be as high as 14.5% [7]. However, data substantiating or refuting this hypothesis remain scarce, underscoring the need for further clinical trials. Endoscopic nasobiliary drainage (ENBD), a widely utilized biliary cooling method, carries an inherent risk of post-procedural acute pancreatitis. This complication is primarily attributed to tissue edema induced by manipulation of the ampulla of Vater, which can transiently obstruct the pancreatic duct. Reported incidence rates of this adverse event reach up to 21% [6]. Given these considerations, additional research is essential to refine biliary cooling strategies, mitigate associated risks, and ensure that liver tumor ablation can be performed safely and effectively.

This study aims to evaluate the safety and efficacy of biliary cooling during thermal ablation of central liver tumors in a Taiwanese patient cohort, providing valuable insights into its role in optimizing oncologic and hepatobiliary outcomes. The findings of this retrospective analysis will contribute to the refinement of treatment protocols, ensuring better preservation of biliary function while maintaining effective tumor eradication.

## 2. Materials and Methods

### 2.1. Participants and Study Design

This study involved a retrospective analysis of a prospective cohort of patients with liver tumors. All patients aged 18 years or older who visited the gastroenterology clinics of Dalin Tzu Chi Hospital and were diagnosed with newly detected liver tumors from July 2020 to June 2023, with available pretreatment and follow-up data, were included. Liver tumors were diagnosed based on imaging examinations such as dynamic contrast-enhanced computed tomography (CT) and magnetic resonance imaging (MRI). A multidisciplinary treatment team comprising radiologists, surgeons, hepatologists, and oncologists evaluated the clinical diagnosis and tumor resectability and discussed the potential benefits, adverse effects, and prognosis of treatment options with patients and their families, after which the patients selected a treatment for liver tumors.

The inclusion criteria were as follows: (1) central liver tumors, (2) patients who underwent thermal ablation, (3) those who received prophylactic bile duct cooling during tumor ablation, and (4) an Eastern Cooperative Oncology Group performance status of 0 or 1. The exclusion criteria included the following: (1) tumors located more than 5 mm away from the central bile duct, (2) combining ablation with percutaneous ethanol injection (PEI) or transarterial chemoembolization (TACE) during the same session, and (3) Child–Pugh class C patients.

“Central liver tumors” are defined based on their proximity to the bile ducts. In the left liver lobe, this includes only the primary duct up to the origin of the duct for segment III. In the right liver lobe, it encompasses the primary duct and its two secondary branches: the right anterior branch serving segments V–VIII and the right posterior branch serving segments VI–VII [6].

### 2.2. Data Collection and Outcome Measures

Clinical details of all patients were recorded retrospectively. Data included age, sex, treatment indication (HCC or colorectal liver metastasis), Child–Pugh class, tumor characteristics (size, tumor–bile duct distance), procedural details, biliary complications (stenosis, abscess, biloma), complications from thermal ablation or ENBD, and treatment outcomes.

Biliary complications after thermal ablation of central liver tumors are defined based on imaging findings [4]. Patients received a contrast-enhanced spiral CT or MRI 1–2 months after liver tumor ablation. A tumor was considered completely ablated if no nodular or irregular enhancement adjacent to the ablative zone was visible in the arterial phase and if an ablative zone margin ≥5 mm from the edge of the tumor was observed in the portal phase [16].

Further follow-up included measurement of serum liver function tests, alpha-fetoprotein, and liver imaging every 3 months. The choice of imaging modality (ultrasound, CT, or MRI) depended on the patient’s clinical condition after discussion by the multidisciplinary treatment team. Local tumor recurrence was defined as new lesions within or at the periphery of the original ablated lesion [17].

At recurrence, treatment decisions were made by a multidisciplinary team. Survival outcomes were reported as overall survival (OS) and progression-free survival (PFS). OS was defined as the time from ablation to death from any cause, loss to follow-up, or data cut-off. Patients lost to follow-up were censored at their last known alive date, and those still alive were censored at the data cut-off (June 30, 2023). PFS was defined as the time from ablation to the first documented tumor progression.

### 2.3. Bile Duct Cooling Technique and Thermal Ablation

**Figure 1:**
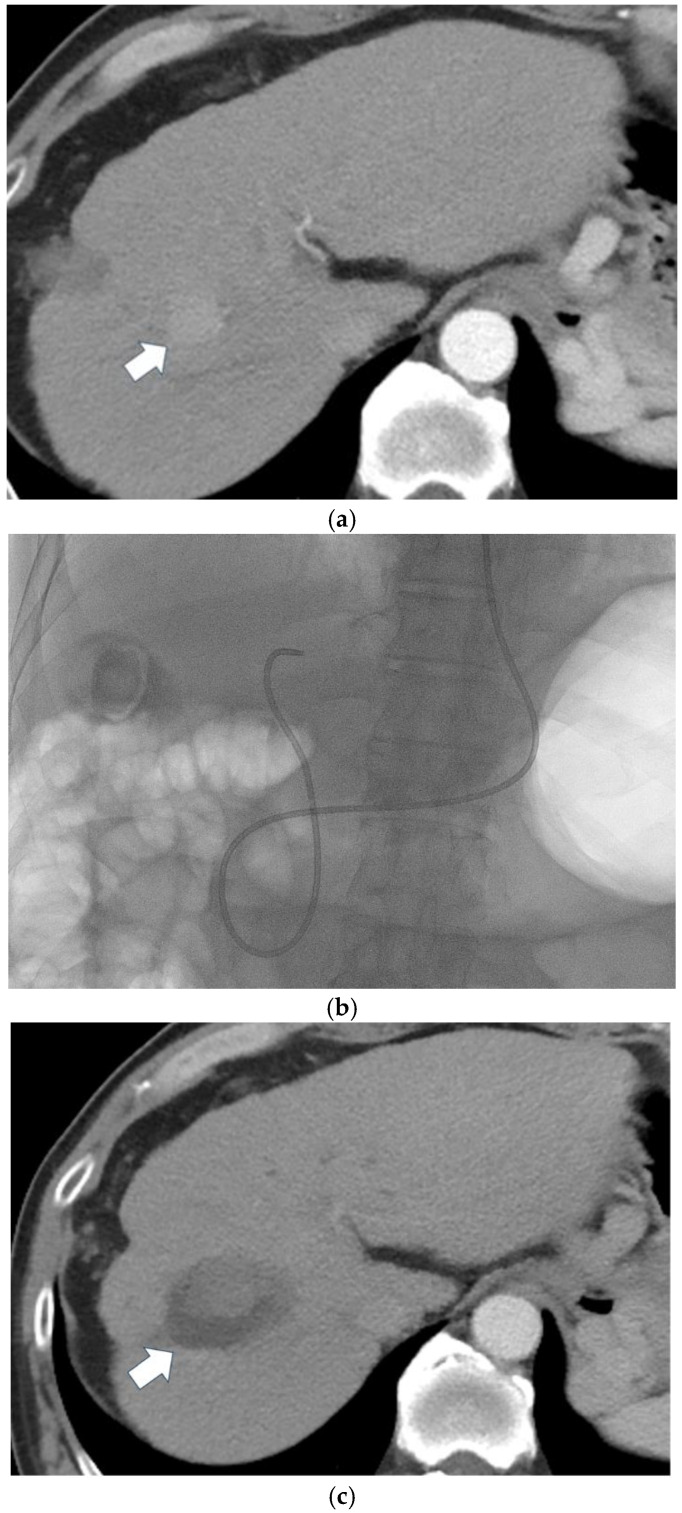
(**a**) Pre-ablation CT of a 67-year-old man showing a 22 mm central HCC in segment 8 (arrow); (**b**) ENBD insertion before ablation; (**c**) post-ablation CT demonstrating complete ablation (arrow).

All study patients received endoscopic retrograde cholangiopancreatography (ERCP) the day before ablation. This scheduling was necessary due to institutional constraints, including the use of separate procedural suites and limited availability of anesthesiology support for both procedures on the same day. Performing ERCP the day prior also allowed sufficient time for post-procedural monitoring to identify any complications before proceeding with thermal ablation. An ENBD tube of 5 to 7.5 Fr was placed with its tip in the subsegmental biliary branch or the common hepatic duct after ERCP (Figure 1). A decompression bag was attached to the ENBD tube to prevent bile from flowing improperly and becoming trapped in the gallbladder. The following day, percutaneous thermal ablation was performed under ultrasound guidance, and 4 °C normal saline was flushed rapidly via the ENBD tube using drip infusion during the procedure.

For tumors measuring less than 3 cm, a 17 G cooled-tip electrode with a 3 cm exposed tip was utilized in one or multiple ablation sessions to achieve a safety margin of 0.5–1.0 cm around the tumor. For tumors exceeding 3 cm, overlapping ablation was conducted using two electrodes with a 3 cm exposed tip or a microwave needle chosen based on the size and shape of the tumor. Temperature and tissue impedance were monitored during the ablation process. The ENBD tube was removed immediately following ablation.

ENBD placement involved contrast injection and the insertion of a tube into the biliary system. Although these procedures were performed under disinfected conditions, they were not strictly sterile. To reduce the risk of major complications such as liver abscess following ENBD, all patients received 1 week of prophylactic antibiotics (third-generation cephalosporin), starting the day before ablation.

### 2.4. Statistical Analysis

Data analysis was performed using IBM SPSS Statistics for Windows, Version 22.0 (IBM Corp., Armonk, NY, USA). Continuous variables were reported as medians with ranges, while categorical variables were presented as numbers with percentages (%).

## 3. Results

We retrospectively reviewed the medical records of 322 patients who underwent thermal ablation during the period from July 2020 to June 2023. Finally, nine patients who had central liver tumors and received prophylactic bile duct cooling during tumor ablation were selected for analysis in this study.

Table 1 shows patient and procedural characteristics. Nine patients (five males, four females) underwent biliary cooling-assisted ablation, with a median age of 67 years (range: 52–82). HCC was the most common indication (67%), followed by colorectal liver metastases (33%). Most patients were Child–Pugh class A (78%), while two patients (22%) were class B. The median tumor size was 27 mm (range: 17–52 mm), and the median tumor–bile duct distance was 1 mm (range: 0–4 mm). RFA was performed in seven patients (78%), while MWA was used in two cases (22%). The diameter size options for the ENBD tube were as follows: 5 Fr in three out of nine cases; 6 Fr in one out of nine cases; 7 Fr in four out of nine cases; and 7.5 Fr in one out of nine cases. The median volume of normal saline infused was 150 mL, with a range from 100 to 200 mL.

Table 2 shows the outcomes of thermal ablation with biliary cooling. The median follow-up was 19 months (range: 3–38 months). All patients achieved complete ablation in one session with no in-hospital mortality. During follow-up, there were no deaths, with a median OS of 19 months (range: 3–38 months) and PFS of 16 months (range: 3–38 months).

No biliary injuries related to thermal ablation were noted. Additionally, other complications associated with thermal ablation with biliary cooling, such as acute pancreatitis, liver abscess, or liver failure, were not observed during the follow-up period. However, one patient (No. 7) developed acute cholangitis after ENBD placement, presenting with fever (38.1 °C) and an increase in serum bilirubin levels from 1.4 to 7.7 mg/dL. The condition was successfully managed with meropenem, allowing RFA to proceed without complications. The patient’s bilirubin levels gradually returned to baseline, and no additional issues, such as liver abscess, were noted during outpatient follow-up.

Two patients (22.2%) experienced tumor recurrence after ablation (Table 2). Patient No. 1 developed infiltrative HCC near the ablation zone 36 months post-treatment. Considering the 36-month interval and the infiltrative characteristics, the recurrence was likely de novo rather than due to incomplete local ablation. Another patient with colorectal liver metastases had multiple recurrences eight months following the procedure. This recurrence may be attributed to the rapid progression of colorectal cancer itself rather than incomplete ablation.

## 4. Discussion

Our study evaluated the impact of biliary cooling with ENBD assistance during thermal ablation for patients with central hepatic tumors. Within our cohort, all patients achieved complete ablation, and none developed biliary injury related to the thermal ablation. Regarding procedural complications, only one patient experienced acute cholangitis related to ENBD drainage.

Although two patients (22.2%) experienced tumor recurrence, neither recurrence was related to incomplete ablation.

Thermal ablation is a safe and effective treatment for liver tumors, but complications can occur. Among them, central bile duct injury is considered a clinically important and serious complication, commonly occurring in patients with pre-existing liver dysfunction [17]. The reported incidence of bile duct injury following thermal ablation varies from 0.5% and 46% per session [6,14,18,19,20], often occurring around the fourth week post-procedure [6,12]. A systematic review and pooled analysis have demonstrated the protective effect of biliary cooling during RFA, reducing the overall rate of biliary complications to as low as 2% for liver lesions near central bile ducts [7]. Although such evidence supports the effect of biliary cooling, most studies have focused on its impact during RFA; however, no studies have specifically investigated its effect on MWA. In this study, we examined the effect of the bile duct cooling technique in patients who underwent thermal ablation, including two patients treated with MWA. None of the patients experienced biliary injury after thermal ablation. Therefore, our results indicate that cold saline infusion through an ENBD tube is a valuable tool that may improve the safety and efficacy of both RFA and MWA for central liver tumors.

In addition to biliary cooling, operators treating central liver tumors may employ the following strategies to achieve curative therapy while minimizing bile duct injury: (1) percutaneous ethanol injection into the tumor region near the bile duct, combined with thermal ablation for the remaining portion; (2) transarterial chemoembolization followed by thermal ablation to target potential residual lesions; and (3) irreversible electroporation [21,22]. However, percutaneous ethanol injection often produces unpredictable outcomes [23], and transarterial chemoembolization frequently leaves residual tumor lesions at the margins, necessitating multiple treatment sessions. While irreversible electroporation is a viable alternative, particularly for liver tumors in high-risk locations, its high cost and limited accessibility significantly restrict its clinical application. Our study results indicate that biliary cooling with ENBD assistance during thermal ablation enables complete ablation in a single session at an acceptable cost.

Endoscopic retrograde cholangiopancreatography (ERCP) with ENBD insertion is a complex endoscopic procedure with associated risks, even when preventive measures are in place [24]. One study reported a 21% incidence of pancreatitis (3 out of 14 patients) linked to nasobiliary tube insertion during ERCP [10]. While no acute pancreatitis cases were observed in our cohort, one patient developed post-ENBD cholangitis, consistent with reported post-ERCP cholangitis rates of 1% to 3% [24]. The insertion of an ENBD tube establishes a direct connection between the biliary system and the external environment, thereby facilitating the migration of enteric flora into the bile ducts. Additionally, the presence of an ENBD tube may compromise bile flow, resulting in bile stasis and subsequent bacterial proliferation. At our institution, thermal ablation is performed on the day following ERCP. It is crucial to administer prophylactic antibiotics [24], ensure adequate bile drainage by attaching a decompression bag, and select a small-caliber ENBD tube to prevent bile flow impairment. Further studies are needed to better understand and mitigate the complications associated with ENBD insertion.

One of the main concerns with RFA in periductal tumors is the “heat-sink” effect caused by biliary cooling. This effect can lead to incomplete ablation and increased recurrence rates. Studies on biliary cooling have assessed local recurrence rates, with a systematic review reporting a 14.5% recurrence at RFA-treated sites [7]. Another study on ENBD-based cooling found no significant difference in recurrence rates between cooling (21%) and non-cooling (23%) groups [9]. Our data confirm these trends, with a 100% complete ablation rate. While two patients (22%) experienced recurrence, neither was due to incomplete ablation. These findings support the role of central bile duct cooling in preventing thermal injury without reducing RFA efficacy.

This study is limited by its retrospective design and small sample size. However, it demonstrates that single-session biliary cooling assistance during thermal ablation is an efficacious and cost-effective approach. These findings contribute to the growing evidence supporting biliary cooling during RFA and MWA. Larger multicenter prospective randomized trials are needed to validate these results and refine strategies for preventing biliary injury during thermal ablation. Secondly, the single-arm design and lack of standardized data archiving limited our ability to compare ablation parameters, such as peak temperature and impedance trends, between patients with and without biliary cooling. Future studies should include real-time data collection and a control group to address this limitation. Third, two patients had a follow-up duration of less than six months. Although the median follow-up for the cohort was 19 months, longer follow-up is needed to better assess long-term efficacy and safety outcomes.

## 5. Conclusions

Cooling the bile duct with cold saline via an ENBD tube during thermal ablation shows promise in preventing bile duct injury in patients with central liver tumors. Our study demonstrated a 100% complete ablation rate with no biliary injuries, emphasizing its potential to enhance procedural safety. However, the risk of ENBD-related complications, such as acute cholangitis, must be considered. Given the study’s small sample size and retrospective nature, further prospective research is needed to confirm these findings and refine biliary cooling techniques for broader clinical application.

## Figures and Tables

**Table 1:** Procedural parameters and baseline patient characteristics in biliary cooling-assisted ablation.

No.	Age (Years)	Gender	Indication	Child-Pugh Class	Tumor Size (mm)	Tumor-Bile Duct Distance (mm)	Method	ENBD Size (Fr.)	Saline Infusion Volume (mL)
1	68	Male	HCV HCC	A	20	0	RFA	7	100
2	69	Male	CLM	A	35	2	RFA	7	NA
3	82	Male	HCV HCC	A	21	1	RFA	6	NA
4	62	Female	HCV HCC	B	38	1	RFA	5	200
5	66	Female	HCV HCC	A	27	4	RFA	7	100
6	65	Male	HCV HCC	A	52	0	MWA	7	NA
7	67	Male	HCV HCC	B	22	2	RFA	7.5	150
8	52	Female	CLM	A	17	0	RFA	5	NA
9	70	Female	CLM	A	40	4	MWA	5	200
Total	67 (52–82)	Male (5, 56%) ^#^	HCC (6, 67%) ^#^	A (7, 78%) ^#^	27 (17–52) *	1 (0–4) *	RFA (7, 78%) ^#^	7 (5–7.5) *	150 (100–200) *
		Female (4, 44%)	CLM (3, 33%) ^#^	B (2, 22%) ^#^			MWA (2, 22%) ^#^		

* median (range); ^#^ (numbers, percentages); ENBD: endoscopic nasobiliary drainage; HCV HCC: hapatiis C virus related hepatocellular carcinoma; CLM: colorectal liver metastases; RFA: radiofrequency ablation; MWA: micorwave ablation; NA: not available.

**Table 2:** Outcomes of Thermal Ablation with Biliary Cooling.

Pt. No.	Duration of Follow-Up (Months)	Biliary Injury	Thermal Ablation Complication	ENBD Complication	Complete Ablation	Local Recurrence/Time (Months)	OS (Months)	PFS (Months)
1	38	No	No	No	Yes	Yes/36	38	36
2	30	No	No	No	Yes	Yes/9	30	9
3	27	No	No	No	Yes	No	27	27
4	26	No	No	No	Yes	No	26	26
5	19	No	No	No	Yes	No	19	19
6	16	No	No	No	Yes	No	16	16
7	14	No	No	Acute cholangitis	Yes	No	14	14
8	6	No	No	No	Yes	No	6	6
9	3	No	No	No	Yes	No	3	3
Total	19 (3–38)	Yes (0, 0%)	Yes (0, 0%)	Yes (1/11%)	Yes (100, 100%)	Yes (2, 22%)	19 (3–38)	16 (3–38)

ENBD: endoscopic nasobiliary drainage; OS: overall survival; PFS: progression-free survival.

## Data Availability

Data are available from the corresponding author upon reasonable request.
